# Microcirculation in open vs. minimally invasive dorsal stabilization of thoracolumbar fractures

**DOI:** 10.1371/journal.pone.0188115

**Published:** 2017-11-15

**Authors:** Bergita Ganse, Miguel Pishnamaz, Philipp Kobbe, Christian Herren, Gertraud Gradl-Dietsch, Franziska Böhle, Bernd Johannes, Bong-Sung Kim, Klemens Horst, Matthias Knobe

**Affiliations:** 1 RWTH Aachen University Hospital, Department of Orthopaedic Trauma, Aachen, Germany; 2 University Duisburg-Essen, LVR Hospital Essen, Essen, Germany; 3 German Aerospace Center (DLR), Institute of Aerospace Medicine, Cologne, Germany; 4 RWTH Aachen University Hospital, Department of Plastic and Reconstructive Surgery, Hand Surgery − Burn Center, Aachen, Germany; University of Illinois at Urbana-Champaign, UNITED STATES

## Abstract

Standard open and percutaneous minimally invasive surgical procedures co-exist in the treatment of fractures of the thoracolumbar spine. Shorter skin incisions just above the pedicles are used in minimally invasive procedures. Full-length skin incisions and invasive preparations are applied in the standard open approach. While both methods show equivalent rates of intraoperative surgical complications and comparable clinical and radiological outcomes, blood loss and operation time have shown to be decreased in minimally invasive treatment. However, no study so far has investigated differences in microcirculation. This study hypothesized less impairment of microcirculation in the minimally invasive approach compared to the open approach and an improvement of microcirculation over time. A prospective cohort study was conducted using non-invasive laser-Doppler spectrophotometry (an O2C “oxygen to see” device) for measurement of cutaneous and subcutaneous blood oxygenation (SO_2_), haemoglobin concentration (Hb), and blood flow at depths of 2, 8, and 15 mm at six locations on the skin. Measurements were performed before surgery, 8 and 24 h after surgery, and 2, 4, 7, 12 and 20 days after surgery, however the number of patients measured decreased towards the later time points. Forty patients were included in the study, 20 with each approach (18 females and 22 males). Pair-wise comparison of the types of surgical procedure for each measurement point revealed a significantly higher flow value in the minimally invasive group at one of the measurement points located between the incisions (P = .041). The point-wise analyses of SO_2_ and Hb did not show significant differences between the approaches. In conclusion, significantly albeit moderately higher flow values could be found in minimally invasive procedures compared to open operations of thoracolumbar fractures in the area of skin that is spared by the incisions.

## Introduction

Fractures of the spine are common and require operative treatment when unstable. Standard open (SO) and percutaneous minimally invasive (MI) procedures co-exist in daily practice. The SO approach requires open preparation of the tissues above the pedicles for screw placement, including a full-length midline skin incision and disconnection of the muscles from their bony attachment to the sides of the spinous processes. The advantages of the MI approach are shorter skin incisions of approximately 2−3 cm each just above the pedicles and penetration through the muscles that stay in place. Rods are inserted from incisions further cranial by penetration through the soft tissue. In the literature, it is assumed that the MI approach minimizes soft tissue injury, blood loss, and pain, as well as complications from decreased blood supply such as infections related to a changed local milieu with local hypoxia and acidosis that decreases the local function of the immune system and thereby supports bacterial growth [1]. Possible advantages of MI over SO have been demonstrated but remain an issue of controversy [2]. While the MI technique is thought to cause less blood loss, pain, and damage to the surrounding tissue, and is also thought to allow for shorter hospitalization and a shorter duration of the surgical procedure, the clinical and radiological outcomes seem to be comparable to the SO approach [3–6]. With regards to wound healing and microcirculation, evidence is rare. MI techniques have been associated with lower infection rates compared to SO approaches in surgical treatment of spinal fractures [7]. Possible causative factors include differences in wound surface size and in the local milieu influenced by inhibited microcirculation [8]. There are, however, no actual data available on differences in local microcirculation comparing the two approaches. The aim of the present study was to compare blood flow, haemoglobin (Hb) concentration, and oxygen saturation (SO_2_) in the area surrounding the site of the surgical procedure in both approaches to evaluate and compare parameters of microcirculation throughout the surgical treatment process over time. Perfusion might be inhibited through the surgical incision after open and minimally invasive surgery but it might recover over time. We hypothesized (1) higher values of tissue oxygen saturation, haemoglobin content and blood flow in the MI approach compared to the SO approach measured at different depth levels and (2) a recovery of microcirculation parameters over time following the healing process.

## Materials and methods

The prospective cohort study was performed at RWTH Aachen University Hospital in Aachen, Germany. Ethics approval was obtained from RWTH Aachen University Hospital IRB (file number EK 007/11). Written informed consent was collected from all participants prior to the start of measurement. The study was conducted according to the Declaration of Helsinki.

### Study design

Patient recruitment took place between December 2010 and December 2011. All patients presenting to the Department of Orthopaedic Trauma at RWTH Aachen University Hospital who matched the inclusion and exclusion criteria were approached. Inclusion criteria were an age of 18 years and older and a spinal fracture between Th10 and L3. Exclusion criteria were pathological fractures, a previous surgical procedure in the measurement area, multiple injuries/polytrauma, severe cardiac or lung disease, immune deficiency, pregnancy, and additional fractures of the spine. All incoming patients with fractures of the spine between Th10 and L3 were screened for the inclusion and exclusion criteria and received information on the study. For those who gave their written informed consent, the surgeon decided whether the SO or MI approach was to be used. The SO procedures were performed using the USS Universal Spine System (Synthes Spine, West Chester, PA, USA). For the percutaneous MI procedures, the Longitude system (Medtronic, Minneapolis, MN, USA) was applied. Four individual experienced surgeons performed the surgeries. All surgeries were performed under general anaesthesia.

### Measurements

Measurements were performed before surgery, 8 and 24 h after surgery, and 2, 4, 7, 12 and 20 days after surgery. A non-invasive laser-Doppler spectrophotometry system was used for measurements (an O2C “oxygen to see” device, LEA-Medizintechnik, Gießen, Germany) to assess cutaneous and subcutaneous SO_2_ (%), Hb (AU), and blood flow (AU) at depths of 2, 8 [9,10], and 15 mm. The measurement volume in which each measurement was performed depended on the tissue density and the used device wavelength, so could not be determined precisely.

**Fig 1:**
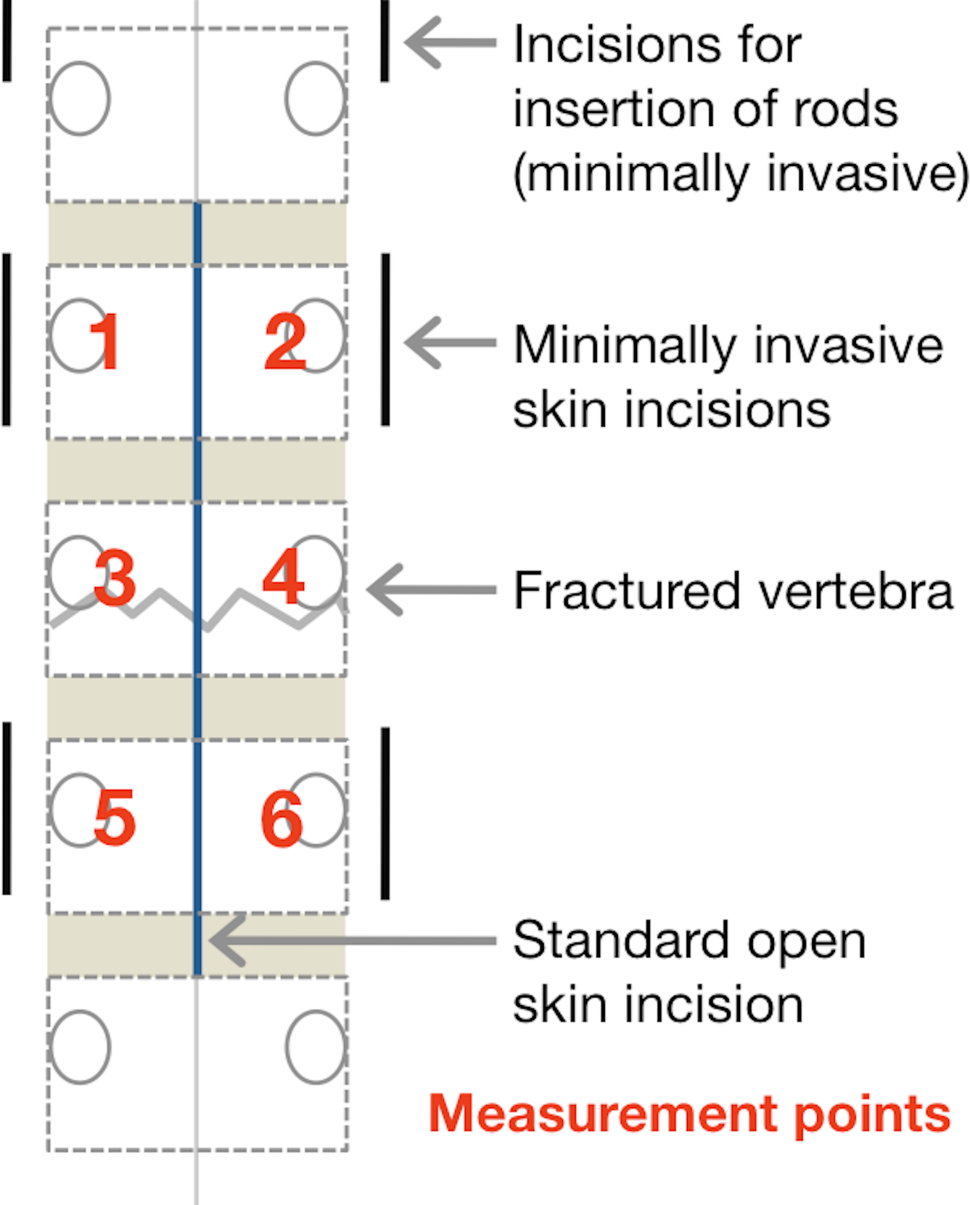
Positioning of the O2C probe and skin incisions.

For all measurements, patients were positioned lying on their left side. Six measurement sites were selected and marked on each patient, independent of the operation type (Fig 1). Each site was located 1.5−2 cm from the midline, above the thoracolumbar muscles, with three on each side. Measurements were taken (1) on top of the pedicles of the vertebra cranial to the fractured vertebra, (2) on top of the pedicles of the fractured vertebra, and (3) on top of the pedicles of the vertebra caudal to the fractured vertebra.

### Data processing and statistical analysis

SPSS (IBM SPSS Statistics release 20.0.0, Armonk, NY, USA) was used to calculate Linear Mixed Effect (LME) models with type of operation, measurement depth, and measurement location as well as their interactions as fixed effects and participants as a random effect (variance components). Significance was assumed at P < .05. Data are presented as counts and as means with SD. Flow values > 1000 AU were an exclusion condition. Hb, SO_2_, and flow were the primary outcome measures of this study. A t-test was conducted to compare groups regarding age, body mass index (BMI), ASA (score of the American Society of Anaesthesiologists), and operation days after fracture.

LME models were chosen for the statistical analysis as many data points were missing in the raw data. It is in the nature of a LME model that an analysis can be performed on incomplete data sets by dealing with missing values better than approaches such as repeated measures analysis of variance.

## Results

### Details on the patient collective

A total of 40 patients (age range 18–87 years, mean age 58.8 +/− 18.8) were included in the study, 20 with the standard open (SO) and 20 with the minimally invasive (MI) surgical approach. Eighteen patients were female (11 SO and 7 MI) and 22 patients were male (9 SO and 13 MI). None of the patients developed a postoperative wound infection. One patient needed revision surgery to correct the positioning of two pedicle screws. No other complications were reported. More details on both groups are shown in Table 1.

**Table 1:** Details on the study participants and comparison of operation groups.

	Standard open (20 patients)	Minimally invasive (20 patients)	P value (t-test)
Mean age	57.0 +/– 18.3	60.6 +/– 19.4	.278
Female/male	11/9	7/13	
BMI	27.2 +/– 4.5 (21–39)	25.4 +/– 4.2 (21–36)	.211
ASA	2.0 +/– 0.6 (1–3)	2.3 +/– 0.8 (1–4)	.272
Smoker	2 (10%)	2 (10%)	
Hypertension	7 (35%)	9 (45%)	
Diabetes mellitus	1 (5%)	2 (10%)	
Days until surgery	2.2 +/– 1.8 (0–6)	3.4 +/– 2.7 (1–11)	.045
Fractured vertebrae	1×Th10, 1×Th11, 6×Th12, 11×L1, 1×L2, 0×L3	0×Th10, 0×Th11, 5×Th12, 8×L1, 5×L2, 2×L3	
AO classification	6×A1, 8×A2, 3×A3, 1×A4, 2×B1	4×A1, 9×A2, 6×xA3, 1×A4	

BMI is defined as the body mass divided by the square of the body height. The ASA physical status classification system was developed by the American Society of Anaesthesiologists and has 5 categories [11]. Fractures were classified according to the AO spine classification system based on pre-operative CT scans [12].

### Differences between operation types

**Fig 2:**
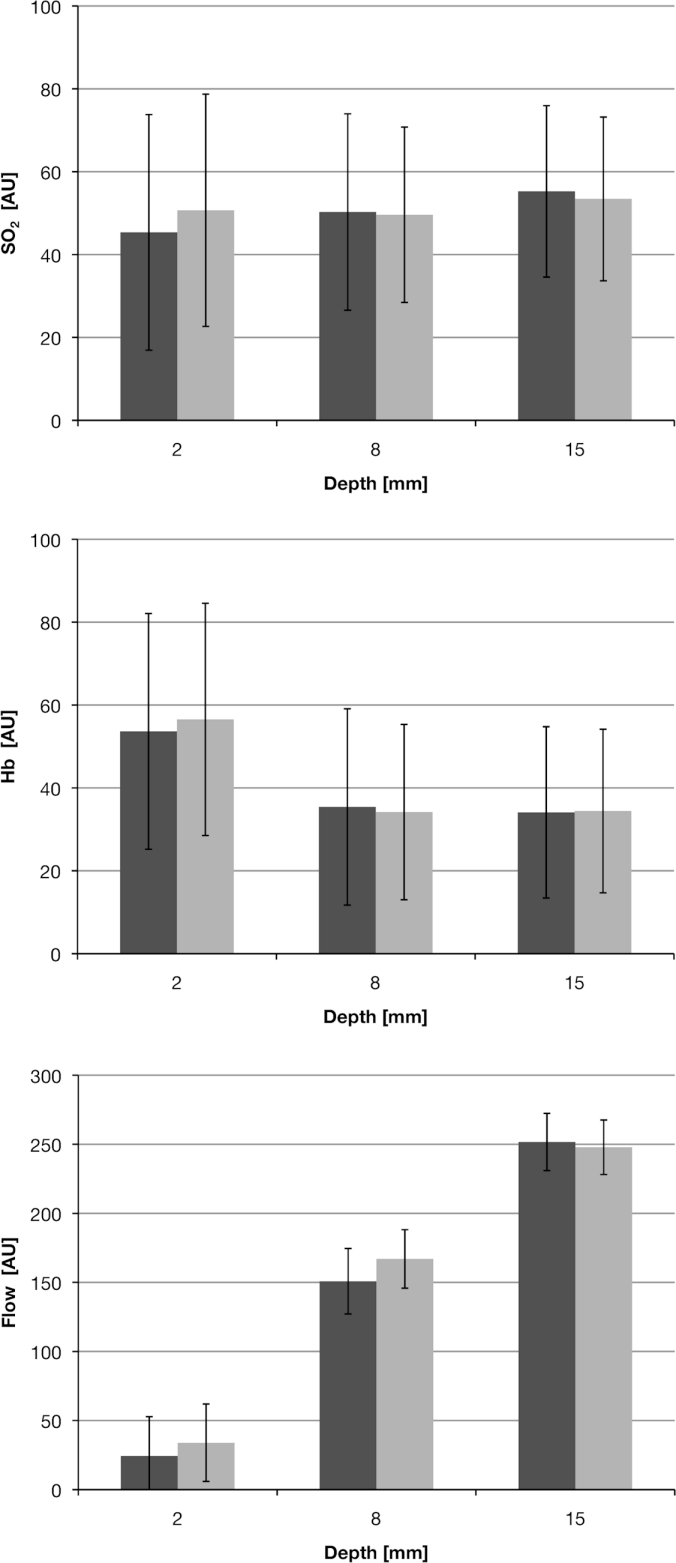
Results over all time and measurement points. Figures show mean values with standard deviation. The standard open approach (SO) is displayed in dark grey, the minimally invasive approach (MI) in light grey. No significant differences were found between operation types in any of the measured parameters arranged by measurement depth. Independent of operation type, the flow differed significantly between depth levels.

Looking at global differences between the two approaches over all time points, measurement points, and depths, there were no significant differences found in any of the measured parameters (SO_2_: P = .579, Hb: P = .291, flow: P = .152). Fig 2 shows these results in depth. Furthermore, a detailed analysis comparing approaches for each measurement point and each parameter revealed significant differences in flow for measurement points 3 and 4 (Table 2). At point 3, flow values were significantly higher for MI compared to SO (mean values and SD in AU, point 3: MI: 160.9 +/– 16.7; SO: 138.7 +/– 28.2). Measurement point 3 is located exactly between the incisions of the MI approach (Fig 1).

**Table 2:** P values for the comparison of operation types.

Measurement point	SO_2_	Hb	Flow
1	.502	.744	.264
2	.564	.587	.417
3	.491	.191	.041*
4	.326	.114	.060
5	.830	.818	.136
6	.930	.077	.924

P values for the pair-wise comparison of operation types (SO vs. MI) for each measured point and measured item.

*significant value with P < .05.

### Changes over time

**Fig 3:**
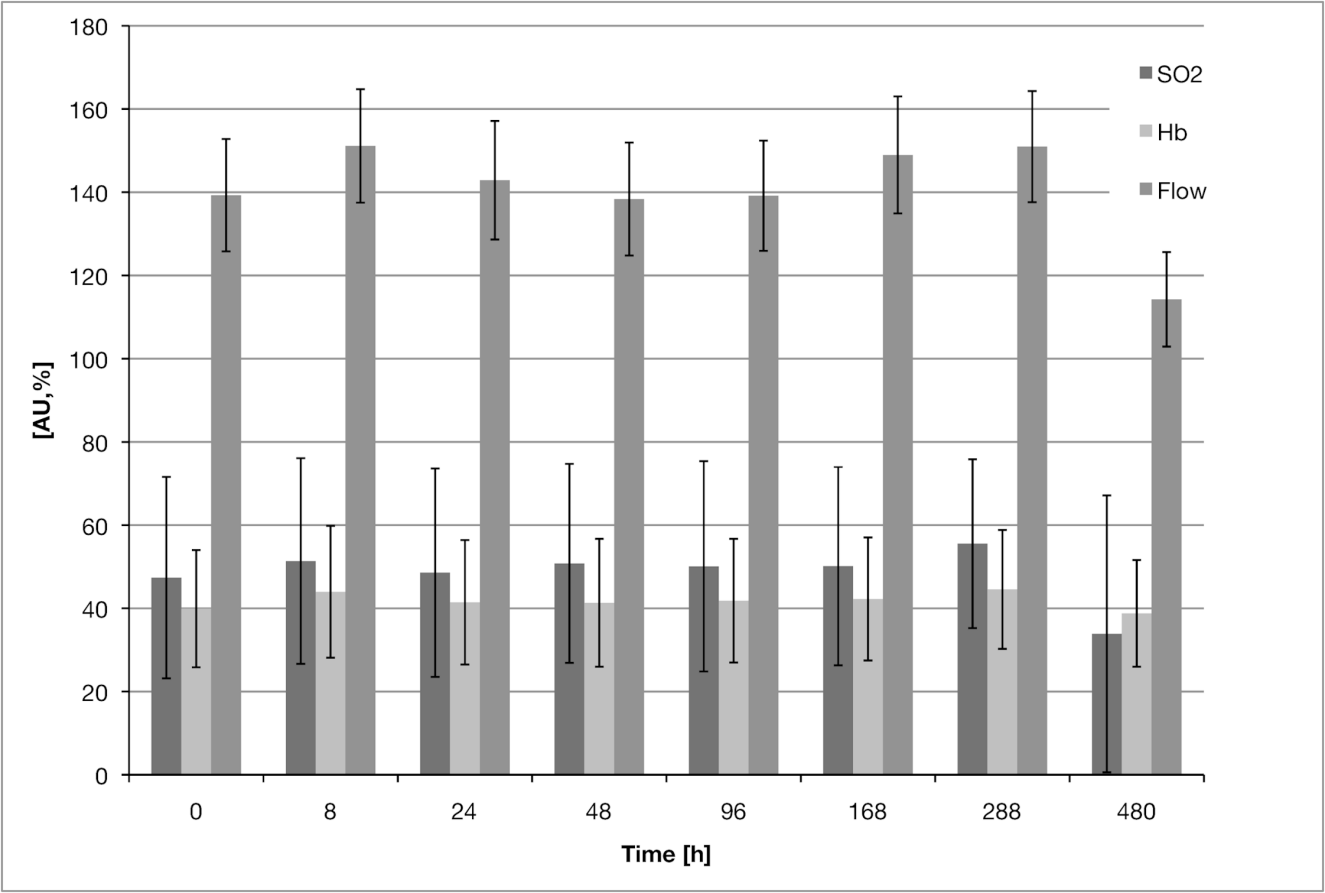
Mean values with pooled measurement points and operation types, showing development over time. SO_2_: %, Hb and flow: AU. The last measurement was performed 20 days after surgery. Time point 0 is the preoperative measurement. See the supplemental raw data for details (Data in S1 File).

No significant changes were found in any of the measured parameters over time. Fig 3 shows the development of mean values over the investigated time span.

## Discussion

A comparison of microcirculation in MI vs. SO operations of thoracolumbar spinal fractures was the aim of the present study. Regarding the hypotheses mentioned in the introduction, our study delivered the following results:

1. A pair-wise comparison of operation types for each measurement point revealed significantly higher flow values for the MI group at point 3, which is between the MI incisions (P = .041). Only local differences were found in flow, but there were no differences found in the pooled data over all measurement points. The point-wise analyses of SO_2_ and Hb did not show significant differences between the approaches. The first hypothesis can therefore not be rejected.
2. No significant changes were found over time in any of the measured parameters. The second hypothesis was rejected.

### Limitations

The possible limitations of the study include a relatively small patient group with mainly healthy individuals. The impact of diseases such as diabetes mellitus could therefore not be studied in the present investigation. A second limitation is that data was collected on each individual for the first measurement points of the study but there were several dropouts for the later measurement times. This lack of data at later times is certainly a disadvantage. Another limitation is the short follow-up time. A longer follow-up period should be considered for future studies.

Regarding limitations of the applied method, it needs to be mentioned that Laser Doppler flowmetry is known to show substantial spatial, temporal, and intra-individual variation [13,14]. Due to the high number of measurement points, depth levels, and time points, it was possible to reduce the impact of this variation by using a LME model for the statistical analysis.

### Comparison of approaches

While equivalent rates of intraoperative surgical complications and comparable clinical and radiological outcomes have been shown in both approaches, blood loss and operation time have presented reduced values in MI procedures [3–5]. Due to smaller skin incisions and less soft tissue damage, the MI approach is thought to allow a better remaining microcirculation. However, in a MEDLINE search, no studies could be found that compare the microcirculation of the SO and MI surgical approaches. This study seems to be the first to do so.

Moderate but significant differences in flow could be found in the detailed analysis of individual measurement points between the MI and SO surgical procedures for spinal fractures. Differences were found in the area between incisions, where the rod is tunnelled through the soft tissue in the MI procedure. At the same time, no significant differences between the two procedures were found in SO2 or Hb. A possible reason is that the blood flow increases in the MI procedure, but due to the increased speed of the erythrocytes passing by, a relevant exchange of oxygen with the surrounding cells does not take place. The unchanged Hb value is most likely caused by the fact that there is no blood stasis in the vessels, and no leakage of plasma. In conclusion, rod insertion through the soft tissue, as done in the MI approach, leads to less impairment of blood flow in the skin compared to the SO approach. As measurements were only taken to a depth of 15 mm, an impaired blood supply in deeper tissue is still possible but most likely not relevant to wound infections. Studies investigating deeper tissues while comparing the MI and SO approaches are rare in the literature. Periosteal vascularisation has previously been investigated in femoral factures by Farouk et al [15]. A silicon injection technique was applied in cadavers to compare SO and MI surgical techniques. The results showed that the MI technique led to better periosteal filling compared to the SO approach group.

### Changes over time

Interestingly, significant changes in microcirculation could not be found over time, not even in the first days of wound healing. Reasons for this result might be found in the physiology of cutaneous blood supply and the structure of blood vessels in the vicinity of wounds. Capillary networks function on a very small scale, and cross-connections of arterioles might be able to compensate structural damage quickly. Furthermore, the plasticity of local capillary structures and the revascularization driven by local hypoxia both act on very short time scales. For example, Lindenblatt et al. [16] investigated revascularisation in a mouse model *in vivo* and found capillary widening in the wound bed on day 1 after grafting, the appearance of capillary buds and sprouts on day 2, and almost complete restoration of the original skin microcirculation on day 5.

### Impact of microcirculation on infection rates

Authors dealing with the reduced infection rates of MI techniques compared to SO surgery have speculated about the relevance of disturbances in microcirculation and the local milieu for the occurrence of infections, and some of their research has shown decreased infection rates with MI approaches [8,17,18].

Our results support these findings and the hypothesis that the MI approach leads to less impairment of microcirculation, possibly resulting in fewer infections. None of the patients in the present study developed a wound infection. Larger groups need to be analysed to get accurate infection rate data for both approaches. Following from our results, MI techniques should be developed in a way that further reduces the length and number of skin incisions to decrease disturbances in microcirculation to an even higher extent. More studies that investigate the local milieu with different techniques to look into local hypoxia and the cellular level would also be of value.

In conclusion, all that the study can really conclude is that the presence of a surgical incision impedes local blood flow (microcirculation) in the vicinity of the incision. The present findings give first evidence for increased blood flow using the MI approach compared to the SO approach in the treatment of thoracolumbar vertebral fractures.

## Supporting information

S1 File Primary data set. This Excel file contains the data used for statistical analysis.
(XLS)
